# Tracing the evolution: the rise of *Salmonella* Thompson co-resistant to clinically important antibiotics in China, 1997–2020

**DOI:** 10.1128/msystems.01018-24

**Published:** 2025-02-12

**Authors:** Weishuai Zhai, Mi Lu, Lanxin Zhao, Pengcheng Du, Shenghui Cui, Yang Liu, Dongmei Tan, Xianying Zeng, Baowei Yang, Ruichao Li, Séamus Fanning, Dejun Liu, Lanqi Li, Xiaoman Zhang, Yang Wang, Li Bai

**Affiliations:** 1 National Key Laboratory of Veterinary Public Health and Safety, College of Veterinary Medicine, China Agricultural University, Beijing, China; 2 Research Unit of Food Safety, Chinese Academy of Medical Sciences, NHC Key Lab of Food, Safety Risk Assessment, China National Center for Food Safety Risk Assessment, Beijing, China; 3 Beijing Key Laboratory of Emerging Infectious Diseases, Institute of Infectious Diseases, Beijing Ditan Hospital, Capital Medical University 12540 , Beijing, China; 4 Department of Food Science, National Institutes for Food and Drug Control 12540 , Beijing, China; 5 Jiangxi Provincial Center for Disease Control and Prevention, Nanchang, China; 6 Institute of Microbe Testing, Guangxi Zhuang Autonomous Region Center for Disease Prevention and Control 12469 , Nanning, Guangxi Zhuang Autonomous Region, China; 7 College of Food Science and Engineering, Northwest A&F University, Yangling, China; 8 Jiangsu Co-Innovation Center for Prevention and Control of Important Animal Infectious Diseases and Zoonoses, Yangzhou University, China; 9 UCD-Centre for Food Safety, School of Public Health, Physiotherapy and Sports Science, University College Dublin, Dublin, Ireland; 10 School of Public Health, The University of Hong Kong, Hong Kong, China; London School of Hygiene & Tropical Medicine, London, United Kingdom

**Keywords:** *Salmonella enterica *serovar Thompson, diarrheal patients, multidrug-resistant, IncC, aquatic product

## Abstract

As clinical *Salmonella enterica* serovar Thompson (*S*. Thompson) emerged among the top ten prevalent serovars in China, understanding the distribution and origin of its multidrug-resistant (MDR) strains becomes imperative. This study employed antimicrobial susceptibility testing, whole-genome sequencing, and bioinformatics analysis to investigate the prevalence and genomic profiles of clinically important *S*. Thompson ST26 across China from 1997 to 2020. Upon analyzing 141 isolates from various sources, we identified 29 isolates, derived from 25 diarrhea patients and four animal-derived foods, displayed co-resistance to ciprofloxacin, cefotaxime, and azithromycin (CIP^R^CTX^R^AZI^R^), all of which are considered the front-line and critically-important antimicrobial agents for treating *Salmonella* infections in humans. The IncC plasmid was the predominant mobile vector identified among the CIP^R^CTX^R^AZI^R^ isolates, harboring four crucial resistance genes *qnrS1*, *qepA4*, *bla*_CMY-2_, and *mph*(A) that confer resistance to three critically important antimicrobials. However, the closely related and clustered IncC-harboring CIP^R^CTX^R^AZI^R^ isolates (0–23 single nucleotide polymorphisms [SNPs]) indicated the clonal spreading of these clinically important isolates in different provinces of China. Notably, the CIP^R^CTX^R^AZI^R^ isolates appeared in aquatic products of animal-derived food, highlighting the possibility of aquaculture practices in the emergence and transmission of important antimicrobial resistance. Our findings emphasize the critical public health implications of IncC-carrying clinically important *S*. Thompson ST26. The study calls for enhanced surveillance of the clinically important *S*. Thompson ST26 clone in clinical and aquaculture and implementation of targeted interventions to mitigate its spread, thereby protecting food safety and public health.

**IMPORTANCE:**

We highlighted the critical veterinary public health issue of clinically important *Salmonella enterica* serovar Thompson (*S*. Thompson) prevalence in animal-derived foods, particularly aquatic products, calling for urgent action. The ability of *S*. Thompson to resist critically important antimicrobials across diverse environments highlights the transmission and survival of resistant strains within the livestock and poultry industry, aquaculture, and food production chains. This study underscores the importance of continuous surveillance of clinically important *S*. Thompson, especially in aquaculture settings, and considers the global trade of aquatic products as a potential vector for international dissemination. Further investigation on the factors contributing to the clone spread of clinically important *Salmonella* strain and the development of intervention strategies to mitigate its public health impact.

## INTRODUCTION

With an annual estimate of 93.8 million infections and approximately 155,000 deaths worldwide, non-typhoidal *Salmonella* (NTS) represents a significant threat to global public health as a leading cause of foodborne illness (1, 2). Among the documented over 2,600 serovars of NTS, *Salmonella enterica* serovar Enteritidis and *S. enterica* serovar Typhimurium are the most frequently reported (3, 4). However, the clinical *S. enterica* serovar Thompson (*S*. Thompson) has recently emerged as a growing concern, now ranking among the top ten prevalent serovars reported in both China and the United States of America (USA) (5, 6). *S*. Thompson has been implicated in various international foodborne outbreaks linked to a diverse range of foods, including seafood, roast beef, and fresh cilantro in the USA (7–9), rucola lettuce in Norway (10), smoked salmon in the Netherlands (11), and chocolate cake in South Korea (12).

Similar to other NTS serovars, infections with *S*. Thompson commonly result in gastroenteritis (11), which is usually self-limiting. However, vulnerable groups such as the elderly, immunocompromised individuals, and children are at risk of developing severe complications like bacteremia and meningitis (2, 13, 14). These cases often require antimicrobial agents to prevent disease escalation and fatalities (14). Currently, the treatments for *Salmonella* infections include three classes of front-line antimicrobial agents: fluoroquinolones (e.g., ciprofloxacin), third-generation cephalosporins (e.g., cefotaxime and ceftriaxone), and macrolides (e.g., azithromycin) (5, 15), though their efficacy is being undermined by increasing resistance rates. Notably, resistance rates of NTS to these antimicrobial agents in China (16.2%, 19.3%, and 7.9%) are higher compared to those in the European Union (EU) (14.1%, 0.8%, and <1.0%) and USA (8.0%, 3.0%, and 1.0%) (5, 15, 16). Furthermore, sporadic reports of concurrent resistance to the three classes of antimicrobial agents in certain serovars, such as in *S. enterica* serovar Kentucky (17), Indiana (18), and Typhimurium (19) have also been reported. In most cases, the genes responsible for conferring bacterial resistance to these antimicrobial agents are independently located either on the chromosome*—*via mutations in *gyrA* and/or *parC* or on transferable plasmids {plasmid-mediated quinolone resistance [PMQR] genes [*qnr* genes, *aac(6')-lb-cr*, *qepA*, and *oqxAB*] and extended-spectrum β-lactamases [such as CTX-M] or macrolide resistance gene *mph*(A)} (20–22). In China, the resistance rates recorded to these antimicrobial agents for clinical *S*. Thompson have exceeded 25%, according to an 8-year study (5). Notably, clinical *S*. Thompson from sporadic cases in some Chinese regions exhibited co-resistance to three front-line antimicrobial agents; however, the extent of the spread of these clinically important *S*. Thompson within China remains to be determined, and the plasmid features and transmission mechanisms of these strains are not fully elucidated (23, 24).

Herein, we report that the co-resistance to ciprofloxacin, cefotaxime, and azithromycin (CIP^R^CTX^R^AZI^R^) in *S*. Thompson is largely due to a single IncC plasmid harboring resistance genes *qnrS1*, *qepA4*, *bla*_CMY-2_, and *mph*(A). Furthermore, clinical *S*. Thompson with this plasmid exhibits a close genetic relationship to those from aquatic sources. This underscores the need for vigilant surveillance of CIP^R^CTX^R^AZI^R^ *S*. Thompson in both aquatic products and human populations via a One Health perspective. Additionally, further genomic epidemiology studies are essential to fully understand the spread and public health impact of CIP^R^CTX^R^AZI^R^ *S*. Thompson.

## MATERIALS AND METHODS

### Bacterial isolates

In our routine surveillance of NTS, a collection of 141 isolates confirmed as *S*. Thompson from multiple Chinese provinces was assembled (Table S1). The highest number of isolates was collected from Guangxi Zhuang Autonomous Region (*n* = 40), followed by Guangdong (*n* = 19), Sichuan (*n* = 16), and Hubei (*n* = 15). Isolates from the remaining regions were fewer than 10 (refer to Table S2 for further details). Of these, 57 clinical isolates were sourced from patients with diarrhea, provided by several regional Centers for Disease Control laboratories in China, facilitated by the State Key Laboratory of Infectious Disease Prevention and Control. These samples originated from nine provinces and were collected between 1997 and 2020. Additionally, 84 isolates were obtained from foods of animal origin. These samples were collected by the National Health Commission Key Laboratory of Food Safety Risk Assessment, spanning 11 provinces and one municipality from 2008 to 2019 (Fig. S1). Clinical fecal samples first underwent enrichment in selenite brilliant green sulfa enrichment broth for 18 h at 36°C ± 1°C, while infection site samples (e.g., blood or cerebrospinal fluid) were cultivated directly on blood agar plates for the same duration and temperature. Isolation and purification of clinical isolates were conducted as per previous methodologies (23). *S*. Thompson from food samples were isolated employing a modified method based on the United States Department of Agriculture-Food Safety and Inspection Service Microbiology Laboratory Guidebook (25). The isolates with typical *Salmonella* phenotypes were all identified on the VITEK 2 COMPACT automated microbial identification platform (bioMérieux, Beijing, China) along with following the Kauffmann-White scheme at the National Health Commission Key Laboratory of Food Safety Risk Assessment in Beijing, China (26).

### Antimicrobial susceptibility testing

We performed antimicrobial susceptibility testing (AST) on all *S*. Thompson isolates using a panel of 14 antimicrobial agents (Table S3), following the standardized broth microdilution method in accordance with the guidelines outlined by the Clinical and Laboratory Standards Institute (CLSI) standard (M100-ED30) (27). The minimal inhibitory concentrations (MICs) of most antimicrobial agents, excluding azithromycin, were interpreted based on the CLSI standard (M100-ED30) (27). For azithromycin, the breakpoint was determined according to the National Antimicrobial Resistance Monitoring System guideline for *Salmonella* (28). The *Escherichia coli* ATCC 25922 and *Staphylococcus aureus* ATCC 29213 (for azithromycin) were included as quality control. Multi-drug resistance (MDR) was defined as resistance to three or more classes of antimicrobial agents.

### Genome sequencing and bioinformatics analysis

Genomic DNA from all *S*. Thompson was extracted using a HiPure Bacterial DNA Kit (Magen, Guangzhou, China), following the standard protocol. The purified DNA was dispatched to Novogene (Beijing, China) for commercial library construction and was sequenced on an Illumina HiSeq 2500 platform, generating read lengths of 150 bp with the paired-end chemistry. One hundred forty-one raw reads were *de novo* assembled using Skesa (v2.4.0) (29). Core genome analysis for all of the 141 nationwide non-repetitive *S*. Thompson genomes was performed using Roary (v 3.13.0) (30), followed by Snippy (v 4.6.0) and Gubbins (v 3.3.0) (31, 32). After that, the phylogenetic tree was generated with Fasttree (v2.1.11) (33), and visualized with Interactive Tree of Life (iTOL) v4 (https://itol.embl.de/). Further bioinformatics analyses involved tools like ResFinder (v4.2.5) (34), PlasmidFinder (v2.0.1) (35), SeqSero (v1.3.1) (36), and MLST (v2.0) (37, 38) were computed using the CGE website with default parameters to identify the antimicrobial resistance (AMR)-encoding genes (including chromosomal point mutations and acquired AMR genes), plasmid replicons, serovars, and sequence types, respectively. Local BLAST searches of *Salmonella* Genomic Island 1 (SGI1) were conducted using BLASTN 2.13.0 (39) with an E-value threshold of 1e-10, employing a reference sequence (accession: AF261825.2) from the NCBI database. Virulence genes were detected by using Abricate (v1.0.1, Github https://github.com/tseemann/abricate) with the database VFDB (Downloaded: July 2023) (40). Additionally, 1,484 publicly available *S*. Thompson genomes were downloaded from the NCBI database (data were accessed in June 2023, Table S4) for extensive comparative phylogenetic analysis, and these primarily originated from North America (*n* = 1,160) and Europe (*n* = 226), with the remainder representing various global regions including China (*n* = 58), South America (*n* = 24), other Asian countries (*n* = 5), and some with unspecified origins (*n* = 11).

### Plasmid conjugation experiments

Plasmid conjugation experiments were conducted using the filter mating method, with sodium azide-resistant *E. coli* J53 as the recipient strain. A mixture of donor strains and the recipient strain J53 (ratio of 1:3) was incubated on LB agar plates for 6 h at 37°C. The successful transconjugants were selected on LB agar plates supplemented with 150 µg/mL sodium azide and 4 µg/mL azithromycin for overnight incubation. Confirmation of transconjugants was achieved through PCR and AST.

### Long-read sequencing and plasmid analysis

For comprehensive genomic characterization, complete genomes of eight representative *S*. Thompson, expressing co-resistance to ciprofloxacin, ceftriaxone, and azithromycin, were sequenced using the Nanopore MinION platform (Oxford, UK). Complete genomes were assembled in a hybrid assembly method with both Illumina and Oxford Nanopore data using Unicycler (v4.0.1) (41). The complete plasmid sequences were extracted using a contig-puller with a 90% minimum identity (https://github.com/kwongj/contig-puller). Gene prediction across the entire genomes and plasmids was annotated using the RAST service (42) and Prokka (v1.14.5) software (43). The AMR determinants, and plasmid replicons located on plasmids were re-identified using the ResFinder (v4.2.5) and PlasmidFinder (v2.0.1), respectively. The putative mobile elements and conjugation-related genes were identified using ISfinder (https://www-is.biotoul.fr/) and oriTfinder (https://tool-mml.sjtu.edu.cn/oriTfinder/oriTfinder.html) with default parameters, respectively. Visualization of single plasmid circular maps was achieved with DNAplotter (v18.0.0) software (44). The alignment of linear plasmids was visualized by Easyfig v2.2.2 (45), and the alignment of circular plasmids with other genomes was produced by BLAST Ring Image Generator (BRIG) v0.95.22 (46).

### Plasmid stability testing

The assessment of plasmid stability commenced with inoculating strains on selective media, ensuring plasmid presence. Post-verification, three individual clones from each strain were cultured in Luria Bertani (LB) medium and incubated overnight at 37°C for growth. Cultures were then diluted (1:1,000 ratio) into 2 mL of fresh LB broth and incubated with shaking at 37°C. Every 3 days, a set of 64 single clones from each strain were selected and cultured on LB agar plates under both selective and non-selective conditions, executed in triplicate. Plasmid stability was quantified based on these experimental results, employing a methodology outlined previously (47).

### Statistical analysis

Correlation analysis of pairwise plasmid and between pairwise plasmid and AMR-encoding genes (ARGs) were calculated using Pearson’s correlation coefficient. To assess the statistical significance of each correlation, *P* values were calculated, and significance levels were marked as *** for *P* < 0.001, ** for *P* < 0.01, and * for *P* < 0.05. The heatmap was generated using the ggplot2 package in R (v 4.1.3), with color gradients indicating the strength and direction of each correlation.

## RESULTS

### *S*. Thompson exhibited widespread distribution and varied AMR profiles

**Fig 1:**
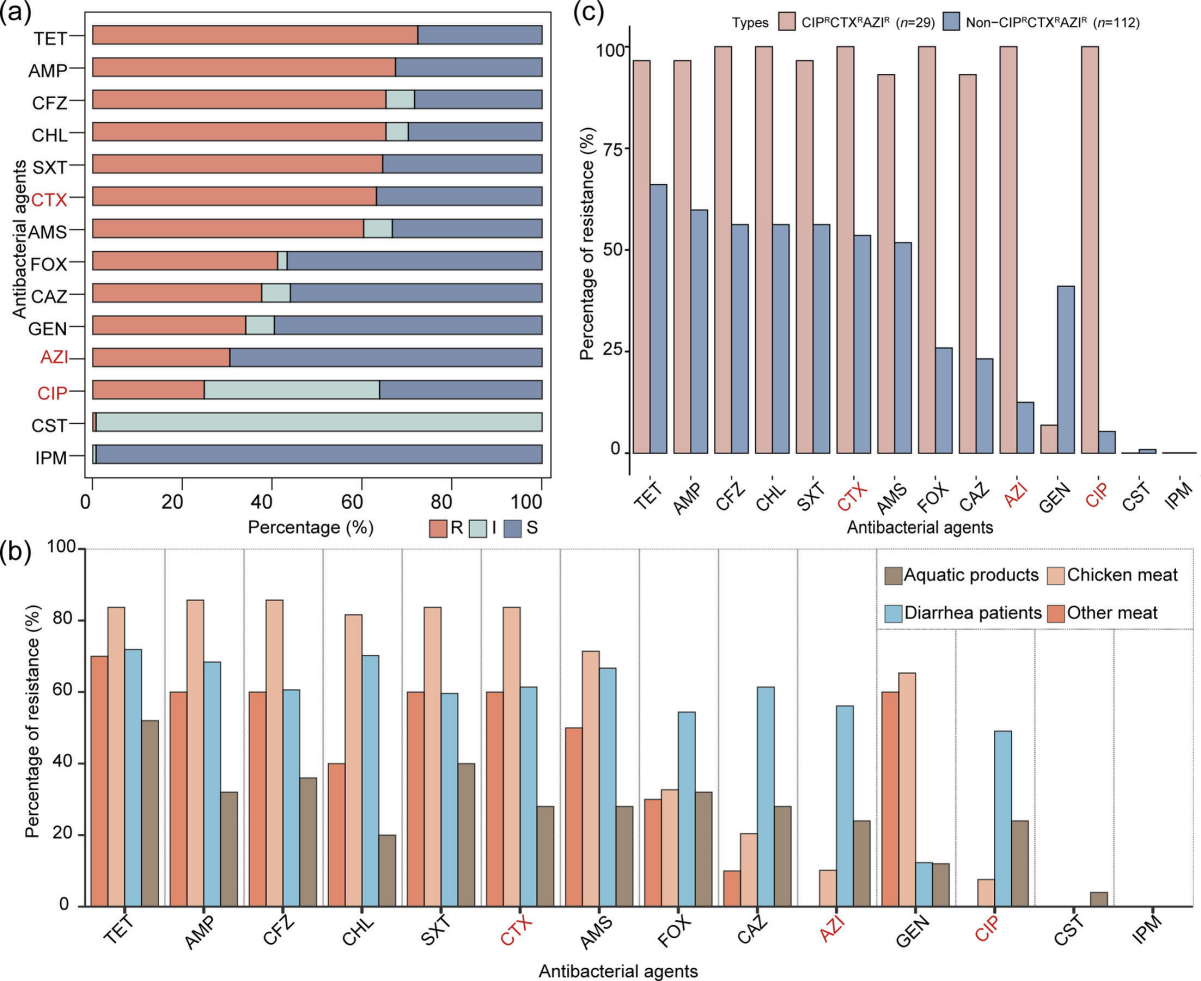
Antimicrobial resistance profile of 141 *S*. Thompson isolates. (**a**) Antimicrobial susceptibility of 141 *S*. Thompson to 14 antimicrobial agents. (**b**) Percentage of resistance observed in *S*. Thompson isolates cultured from different sources. (**c**) Comparative analysis of resistance rates between CIP^R^CTX^R^AZI^R^ *S*. Thompson and other isolates. R, resistant; I, intermediary; S, susceptible; TET, tetracycline; AMP, ampicillin; CFZ, cefazolin; CHL, chloramphenicol; SXT, trimethoprim/sulfamethoxazole; CTX, cefotaxime; AMS, ampicillin/sulbactam; FOX, cefoxitin; CAZ, ceftazidime; AZI, azithromycin; GEN, gentamycin; CIP, ciprofloxacin; CST, colistin; IPM, imipenem.

Our study comprised some 141 *S*. Thompson collected over an extended time period: 57 from clinical samples cultured from diarrheal patients (1997–2020), 84 from animal-derived foods (aquatic products *n* = 25, chicken meat *n* = 49, and other meat *n* = 10, 2008–2019). Using whole-genome sequencing (WGS) data, all isolates were identified by *in silico* MLST and serotyping as *S*. Thompson ST26. Antimicrobial susceptibility analysis exhibited varying resistance rates: 72.3% (102/141) to tetracycline, 67.4% (95/141) to ampicillin, 65.2% (92/141) to chloramphenicol, 60.3% (85/141) to ampicillin/sulbactam, 65.2% (92/141) to cefazolin, and 64.5% (91/141) to trimethoprim-sulfamethoxazole. Notably, resistance to colistin was rare at 0.7% (1/141), and no resistance to imipenem was detected (Fig. 1a). An MDR profile was observed in 100 (70.9%) of the study isolates. Chicken meat-derived isolates demonstrated higher resistance rates (ampicillin, tetracycline, chloramphenicol, ampicillin/sulbactam, cefazolin, cefotaxime, trimethoprim-sulfamethoxazole), ranging from 71.4% (ampicillin/sulbactam, 35/49) to 85.7% (ampicillin, 42/49), compared to aquatic product-derived isolates, which ranged from 20.0% (chloramphenicol, 5/25) to 52.0% (ampicillin, 13/25) (Fig. 1b).

**Fig 2:**
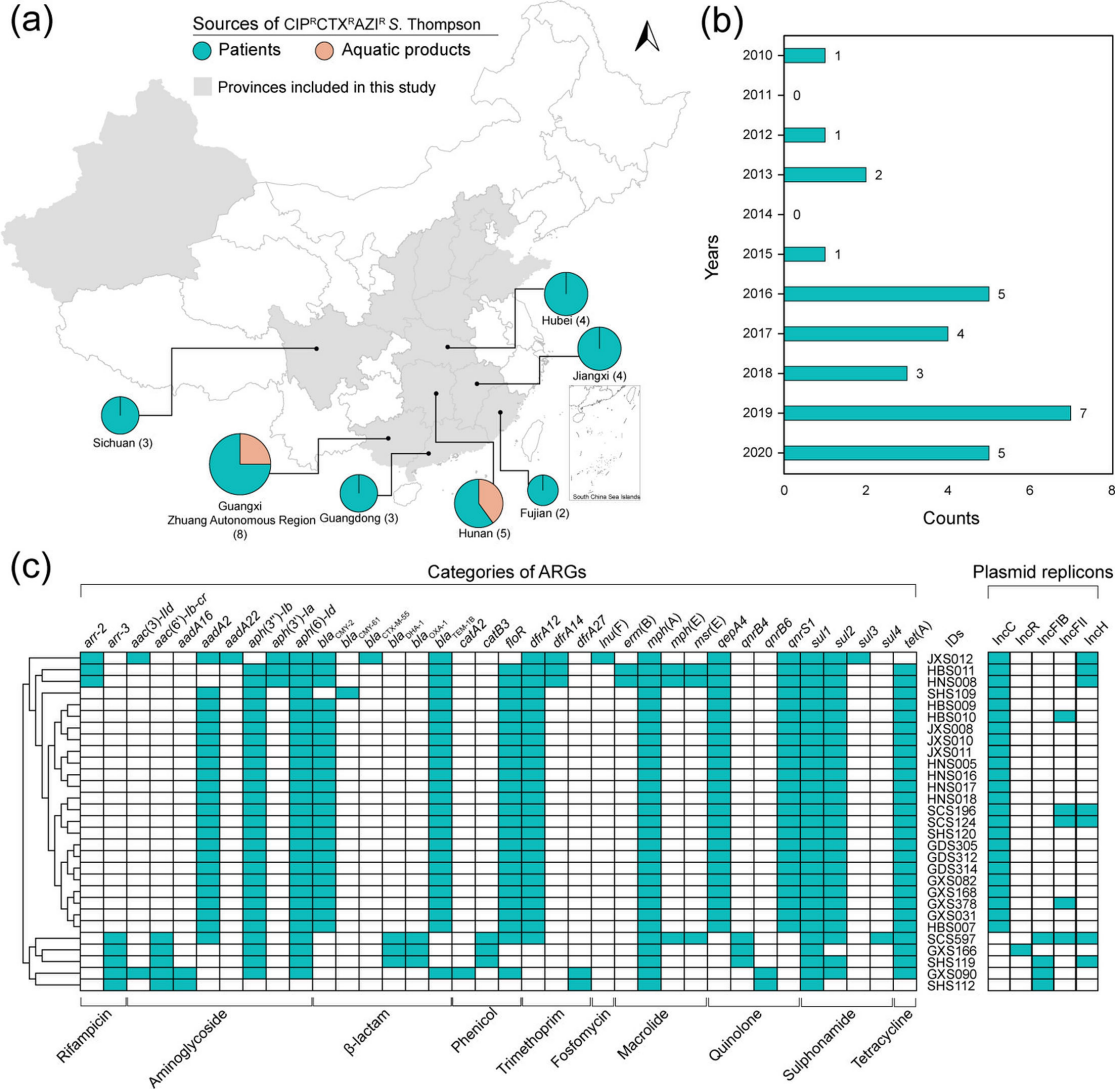
Comprehensive analysis of 29 CIP^R^CTX^R^AZI^R^ *S*. Thompson: geographic distribution, temporal trends, and genetic characteristics. (**a**) Geographic distribution of 29 CIP^R^CTX^R^AZI^R^ *S*. Thompson. The gray background represents the collection regions for all 141 *S*. Thompson, the size of the pies represents the numbers of *S*. Thompson, and the colors of the pies represent the sources of strains. The map image was produced with QGIS 3.8. (**b**) Distribution of CIP^R^CTX^R^AZI^R^ *S*. Thompson from 2010 to 2020. (**c**) Heatmap of ARGs and plasmid replicons in 29 CIP^R^CTX^R^AZI^R^ *S*. Thompson.

One hundred six (75.2%) isolates expressed resistance to at least one of the three front-line antimicrobials. Specifically, resistance was observed in 89 (63.1%) isolates to cefotaxime, 43 (30.5%) to azithromycin, and 35 (24.8%) to ciprofloxacin. Co-resistance to these antimicrobial agents, defining the CIP^R^CTX^R^AZI^R^, was found in 29 (20.6%) *S*. Thompson isolates, which showed significantly higher resistance rates to most tested compounds, except for gentamicin, colistin, and imipenem, compared to the other 112 (79.4%) *S*. Thompson isolates (Fig. 1c). The CIP^R^CTX^R^AZI^R^ expressing isolates originated from diverse sources: 25 from diarrhea patients, four from aquatic products, and were distributed across various regions in China (Fig. 2a), including Eastern (Fujian and Jiangxi), South (Guangxi Zhuang Autonomous Region and Guangdong), Central (Hunan and Hubei), and Southwest (Sichuan) from 2010 to 2020 (Fig. 2b).

### The AMR in *S*. Thompson was driven by corresponding antimicrobial resistance genotypes

To identify the determinants that contribute to the resistance phenotype, we analyzed ARGs in 29 CIP^R^CTX^R^AZI^R^ *S*. Thompson through WGS. Some 36 distinct acquired ARGs conferring resistance to 10 classes of antimicrobial agents (Fig. 2c) were recorded. The most prevalent resistance genes, *sul1* and *mph*(A), were found in all isolates, followed by *aph*(6)*-Id* in 28 isolates, and *aph*(*3''*)*-Ib*, *sul2*, and *tet*(A) in 27 isolates each. PMQR genes associated with CIP^R^ exhibited diversity, with *qnrS1* and *qepA4* detected in 24 isolates, *and aac*(6’)*-Ib-cr* and *qnrB* (specifically *qnrB4* in three isolates and *qnrB6* in two isolates) identified in the remaining five isolates (Fig. 2c). Additionally, all 29 isolates carry a *parC* mutation (giving rise to the T57S substitution), which does not confer CIP resistance. Instead, this mutation has been associated with increased sensitivity to ciprofloxacin, potentially acting as a compensatory change, particularly in isolates with concurrent mutations in *gyrA* (48, 49). Several β-lactamase genes such as *bla*_CMY-2_ (*n* = 23) along with *bla*_TEM-1B_ (*n* = 25) were commonly observed, along with *bla*_OXA-1_ (*n* = 3), *bla*_DHA-1_ (*n* = 3), *bla*_CTX-M-55_ (*n* = 1), and *bla*_CMY-61_ (*n* = 1). Notably, the *mph*(A) gene associated with AZI^R^ was present in all isolates, with three of these also carrying *msr*(E) or *erm*(B). Plasmid replicon analysis identified a range of Inc-type plasmids, predominantly IncC (*n* = 24), followed by IncH (*n* = 7), IncFII (*n* = 5), IncFIB (*n* = 4), and IncR (*n* = 1) (Fig. 2c). The multidrug-resistant gene cluster SGI1 was not detected in all analyzed isolates.

### Clonal transmission and global spreading potential of CIP^R^CTX^R^AZI^R^ *S*. Thompson

**Fig 3:**
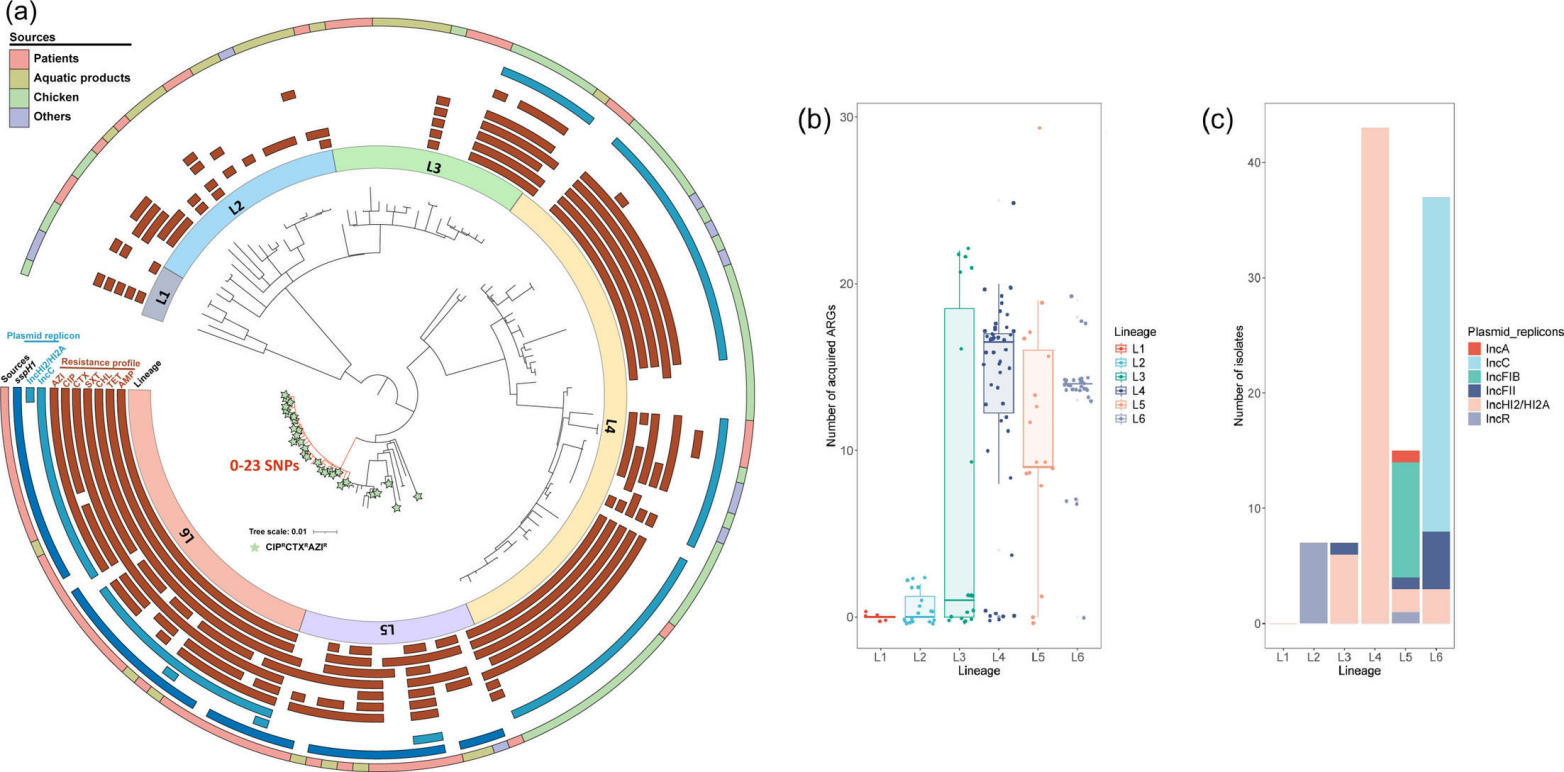
Phylogenetic analysis and distribution of antimicrobial resistance profiles and plasmid replicons identified in 141 *S*. Thompson in China. (**a**) Phylogenomic relationship among 141 analyzed *S*. Thompson genomes. The orange branch represents the lineage containing isolates that belong to lineage L6. (**b**) Distribution of ARG quantities carried by *S*. Thompson across different lineages. (**c**) Distribution of the six plasmid replicons across different lineages.

To further explore the relationship among 29 CIP^R^CTX^R^AZI^R^ *S*. Thompson, we constructed a phylogenetic tree from the core-genome SNP of 141 *S*. Thompson and categorized them into six distinct lineages: L1 (*n* = 5), L2 (*n* = 19), L3 (*n* = 20), L4 (*n* = 50), L5 (*n* = 17), and L6 (*n* = 30) (Fig. 3a). Notably, the MDR isolates predominantly clustered in lineages L4, L5, and L6 (Fig. 3b). These lineages displayed varied AMR profiles: e.g., L4 isolates expressed resistance to cefotaxime and traditional front-line drugs, including ampicillin, chloramphenicol, and trimethoprim-sulfamethoxazole, but not commonly to ciprofloxacin and azithromycin. Besides, over half of the isolates in L6 were resistant to most of the tested antimicrobial compounds, including cefotaxime, ciprofloxacin, and azithromycin. Similarly, the CIP^R^CTX^R^AZI^R^ *S*. Thompson clustered mainly in L6 (80.0%, 24/30) and L5 (29.4%, 5/17). These L6 isolates shared a close genetic relationship showing a difference of 0–23 SNPs (Fig. 3a; Table S5), suggesting possible clonal expansion of CIP^R^CTX^R^AZI^R^ *S*. Thompson across diverse environments. Plasmid distribution varied widely across lineages, with the IncFIB replicon exclusively found in L5 (58.8%, 10/17), while IncC was present in L6 (96.6%, 29/30). The first recorded case of CIP^R^CTX^R^AZI^R^ *S*. Thompson carried IncC isolated SHS109 and SHS120 from diarrhea patients in 2013 in Guangxi Zhuang Autonomous Region and Fujian, with annual detections thereafter up to 2020 (except 2014). The IncHI2/HI2A replicon (*n* = 54) was widely distributed in four lineages (L3, L4, L5, and L6) but predominantly in lineage L4 (*n* = 43) (Fig. 3c). In-depth analysis of virulence factors pinpointed SspH1, a critical E3 ubiquitin ligase that plays a role in influencing host cell signaling, especially targeting NF-κB to modulate immune responses and promote the invasion and survival of *Salmonella* within host cells (50, 51). This virulence factor was predominantly identified in bacterial isolates clustering in L5 (82.4%, 14/17) and L6 (93.3%, 28/30) (Fig. 3a; Table S6), suggesting its critical role in the pathogenicity and spreading of CIP^R^CTX^R^AZI^R^ *S*. Thompson. Additionally, the isolates from clinical settings and animal-derived foods spanned all lineages. The chicken-derived isolates belonged to L4 (74.0%, 37/50), L1 (60.0%, 3/5), L3 (35.0%, 7/20), and L2 (10.5%, 2/19), those from aquatic products were found in L2 (47.4%, 9/19), L3 (35.0%, 7/20), L5 (29.4%, 5/17), and L6 (10.0%, 3/30), L4 (2.0%, 1/50) (Table S1). The majority of isolates from aquatic products in L5 and L6 were linked to fish (*n* = 5).

In comparing our 141 *S*. Thompson with an additional 1,484 *S*. Thompson ST26 from the NCBI database, we distinguished two new lineages, termed Lineage A and Lineage B. Lineage A encompassed the majority of strains from China, whereas Lineage B mainly included strains from North American countries, such as the USA. All strains identified as L5 and L6 in our study were classified under Lineage A, indicating these CIP^R^CTX^R^AZI^R^ strains likely originated and evolved in China. Notably, we observed a close phylogenetic relationship between 19 publicly available IncC-carrying *S*. Thompson strains from China (11 from patients and eight from turtles) and our 30 isolates of lineage L6, further underscoring the role of aquatic products in the dissemination of CIP^R^CTX^R^AZI^R^ *S*. Thompson. Additionally, certain clinical strains from the United States (*n* = 4) and the United Kingdom (*n* = 1) also showed close phylogenetic ties to L6 strains, with four of these isolates carrying the IncC plasmid, indicating the potential for international transmission of CIP^R^CTX^R^AZI^R^ IncC-carrying *S*. Thompson (Fig. S2).

### IncC plasmids conferring CIP^R^CTX^R^AZI^R^ can transfer among *S*. Thompson

Despite the emergence of co-resistance to ciprofloxacin, cefotaxime, and azithromycin, it remains unclear whether their determinants were located on transferable plasmids or not. To validate the transferability of CIP^R^CTX^R^AZI^R^ among these *S*. Thompson, conjugation experiments were then performed on randomly selected *S*. Thompson carrying IncC from lineage L6 (SHS120, HBS007, HBS009, HBS011, JXS008, JXS011, GXS168, GXS031, GXS082), and five isolates carrying various non-IncC replicons from L5 (GXS090, GXS166, SCS597, SHS112, SHS119). All isolates carrying IncC replicon successfully transferred their resistances to ciprofloxacin, cefotaxime, and azithromycin to the recipient, *E. coli* J53, simultaneously, with frequencies ranging from 10^−8^ to 10^−6^. The MICs of the transconjugants against ciprofloxacin, cefotaxime, and azithromycin increased from 0.015 µg/mL to 8–16 µg/mL, and 0.03 µg/mL to ≥4 µg/mL, and 1 µg/mL to 16–128 µg/mL, respectively. In addition, among the five isolates carrying non-IncC replicons, only one isolate (SHS119), which harbored the IncFIB/HI2/HI2A plasmid replicon, was able to simultaneously transfer resistance to ciprofloxacin (from 0.015 µg/mL to 1 µg/mL), cefotaxime (from 0.03 µg/mL to 1 µg/mL), and azithromycin (from 1 µg/mL to 16 µg/mL) to the recipient (Table S7).

### The conjugative IncC plasmids conferring CIP^R^CTX^R^AZI^R^ remained conserved

**Fig 4:**
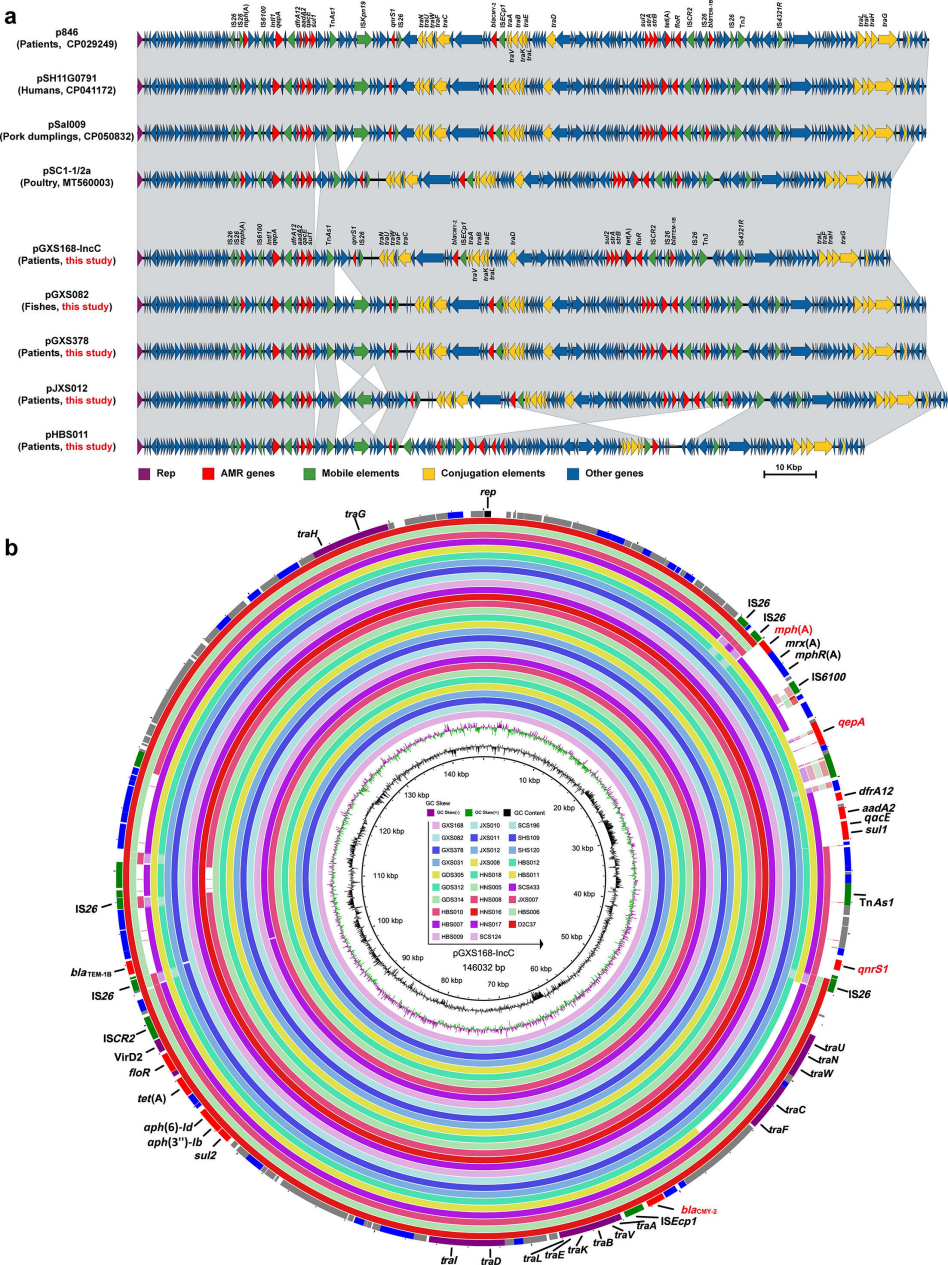
Multiple sequence alignment of IncC plasmids in *S*. Thompson. (**a**) Sequence alignment of five local IncC plasmids and four publicly available IncC plasmids of *S*. Thompson. (**b**) Sequence alignment of pGXS168-IncC to other IncC-carrying *S*. Thompson strains in lineage L6.

To further verify the location of CIP^R^CTX^R^AZI^R^ determinants, we randomly sequenced five isolates from L6 carrying IncC replicon using the nanopore sequencing platform and recorded that the CIP^R^CTX^R^AZI^R^ genes were located on IncC plasmid in all five isolates. These five plasmids, with sizes ranging from 141- to 157-kbp, were highly conserved and featured minor variations due to IS elements. Critically, they harbored 13 ARGs, including *qnrS1*, *qepA4*, *bla*_CMY-2_, and *mph*(A) conferring resistances to ciprofloxacin, cefotaxime, and azithromycin, respectively (Fig. 4a). Taking plasmid pGXS168-IncC, one of the five plasmids, for example, the *bla*_CMY-2_ gene was located adjacent to IS*Ecp1* in the conjugative transfer region of the conserved plasmid backbone. The remaining 12 ARGs were located on two multidrug resistance regions (MDRRs, Fig. S3). MDRR1 contained six ARGs, including *bla*_TEM-1B_ (β-lactam), *floR* (phenicol), *tet*(A) (tetracycline), *aph*(3'')-*Ib* and *aph*(6)*-Id* (aminoglycoside), *sul2* (sulfonamide), and MDRR2 contain other six ARGs, including *qnrS1* and *qepA4* (fluoroquinolone), *mph*(A) (macrolide), *aadA2* (aminoglycoside), *sul1* (sulfonamide), *dfrA12* (trimethoprim).

To explore whether these conserved plasmid sequences were distributed among IncC plasmid-harboring CIP^R^CTX^R^AZI^R^ *S*. Thompson, we compared the genome sequence of all isolates carrying IncC plasmid with plasmid pGXS168-IncC using BLASTn (Fig. 4b). This analysis confirmed that all isolates shared highly similar sequences with the latter. A subsequent BLASTn search against the NCBI nr database yielded four complete plasmids (p846 [Accession: NZ_CP029249], pSH11G0791 [NZ_CP041172], pSal009 [NZ_CP050832], and pSC1-1/2a [NZ_MT560003]) with perfect nucleotide sequence identity and coverage to plasmid pGXS168-IncC. These plasmids all recovered from *S*. Thompson, which derived from humans (*n* = 2), poultry (*n* = 1), and animal-derived food (*n* = 1) in China (Fig. 4a). Notably, no plasmids closely resembling plasmid pGXS168-IncC were found in bacterial species other than *S*. Thompson within the NCBI database. Although a similar IncC plasmid (~155–168 kbp) in identified *E. coli*, *Klebsiella pneumoniae,* and *Proteus mirabilis* exhibited 100% nucleotide identity and >75% coverage with plasmid pGXS168-IncC, most of these plasmids lacked the six AMR genes, including *qnrS1*, *qepA*, and *mph*(A), present in MDRR2 (Fig. S4 and S5). This suggests a unique formation of clinically important IncC plasmids in *S*. Thompson. Further investigation into plasmid-host interactions through stability tests of two randomly selected IncC-carrying isolates revealed their high stability over 28 days of subculturing.

### Non-IncC plasmids in CIP^R^CTX^R^AZI^R^ *S*. Thompson

**Fig 5:**
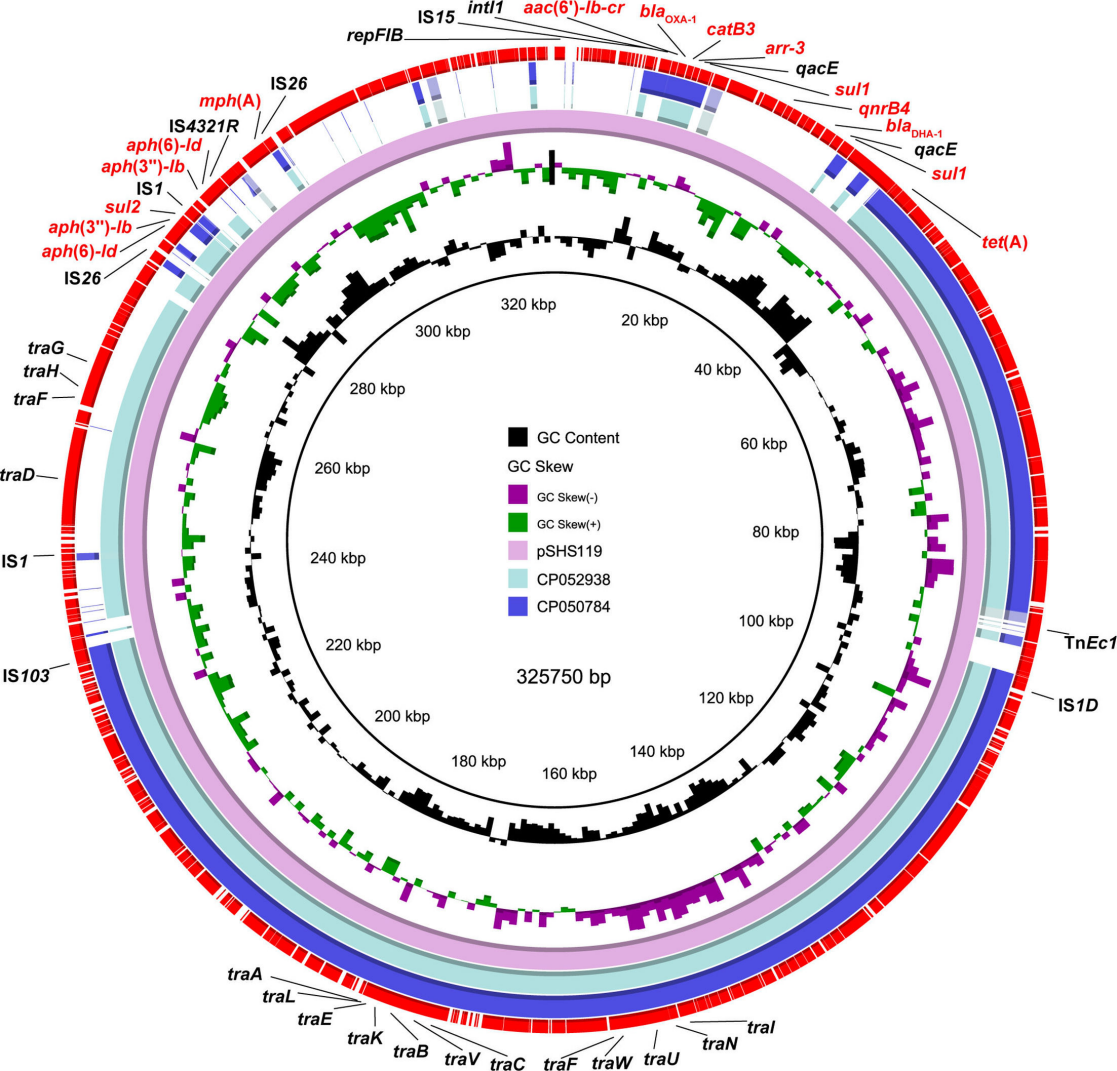
Comparative sequence analysis of IncFIB/HI2/HI2A plasmid pSHS119.

In addition to the IncC plasmid, we also analyzed the genetic characteristics of one IncFIB/HI2/HI2A type hybrid plasmid conferring CIP^R^CTX^R^AZI^R^. The IncFIB/HI2/HI2A pSHS119 was 326 kbp in size and had a GC content of 46.9%. It contained 12 ARGs, including *aac*(6')*-Ib-cr*, *qnrB4*, *bla*_DHA-1_, *bla*_OXA-1_, *mph*(A), *arr-3*, *aph*(3'')*-Ib*, *aph*(6)*-Id*, *catB3*, *sul1*, *sul2*, and *tet*(A). Plasmid pSHS119 exhibited over 99% nucleotide sequence identity and 63%–74% coverage to two plasmids (273 kbp pK16SI097, CP052938 and 255 kbp pSI67-1, CP050784) from chicken-derived *S. enterica* serovar Indiana (Fig. 5).

### Correlation of two plasmid replicons and 31 ARGs in *S*. Thompson

**Fig 6:**
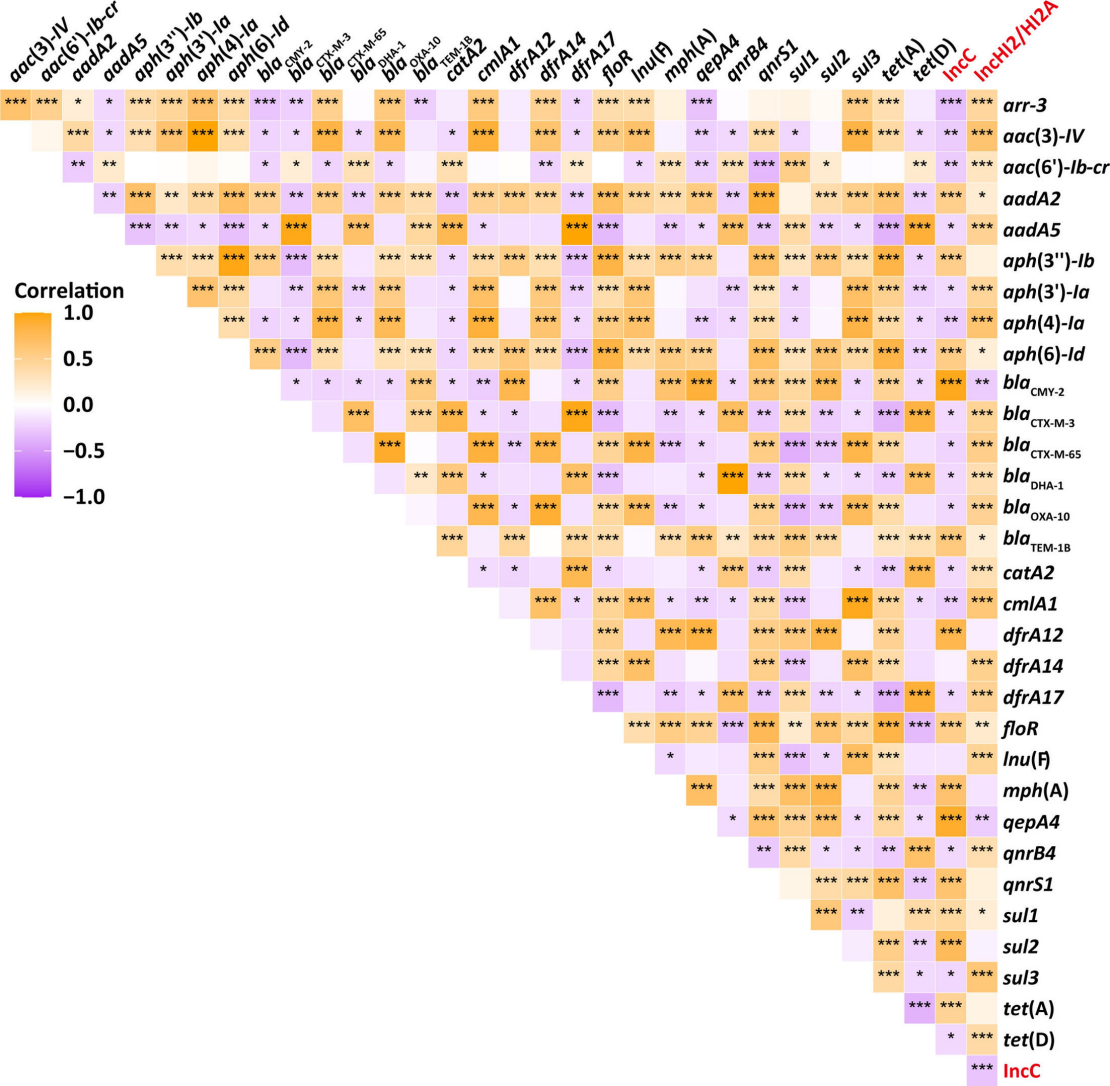
Correlation between ARGs and plasmid replicons. The x axis and y axis represent 31 ARGs and two plasmid replicons, IncC and IncHI2/HI2A. The heatmap displays Pearson’s correlation coefficients (r-values), with orange indicating positive and purple indicating negative correlations. Significance levels are denoted as *P* < 0.05 (*), *P* < 0.01 (**), and *P* < 0.001 (***).

Subsequently, we analyzed the co-occurrence of 31 resistance genes on IncC and IncHI2/HI2A plasmid replicons. The results revealed that IncC plasmid replicon was positively associated with the following 13 ARGs: *bla*_TEM-1B_ and *bla*_CMY-2_ (β-lactam), *floR* (chloramphenicol), *tet*(A) (tetracycline), *aph*(3")-*Ib* and *aph*(6)-*Id* (aminoglycoside), *sul2* (sulfonamide), *qnrS1* and *qepA4* (fluoroquinolone), *mph*(A) (macrolide), *aadA2* (aminoglycoside), *sul1* (sulfonamide), and *dfrA12* (trimethoprim). This correlation aligns with the ARGs previously described as being carried by IncC plasmid pGXS168-IncC. The IncHl2/HI2A plasmid replicon was found to be significantly associated with more ARGs than the IncC replicon, including *bla*_CTX-M-3_, *bla*_CTX-M-65_, *bla*_DHA-1_, and *bla*_OXA-1_ (β-lactam), *tet*(D) (tetracycline), *aac*(3)-*IV*, *aac*(6')-*Ib-cr*, *aph*(3')-*Ia*, *aph*(4)-*Ia*, and *aadA5* (aminoglycoside), *catA2*, *cmlA1* (chloramphenicol), *dfrA14* and *dfrA17* (trimethoprim), *qnrB4* (fluoroquinolone), *sul3* (sulfonamide), *arr-3* (rifamycin), and *lnu*(F) (lincosamide) (Fig. 6).

## DISCUSSION

The global prevalence of *S*. Thompson is on the rise, elevating it to a critical public health issue. This serovar has moved from the 16th to the 10th most important bacterial pathogen, highlighting a 79.2% increase from 2006 to 2016 (6) and causing recent outbreaks in the USA (7). In China, *S*. Thompson has been sporadically detected in patients (5), animals (52), meats (53, 54), and aquaculture products (55, 56) across multiple provinces over the past decade. Notably, the recent emergence of CIP^R^CTX^R^AZI^R^ *S*. Thompson in diarrheic patients from Shanghai, Guangxi Zhuang Autonomous Region, and Hunan provinces has raised significant health concerns (23, 24, 57). Considering the foodborne nature of *Salmonella*, controlling CIP^R^CTX^R^AZI^R^ *S*. Thompson in humans necessitates a holistic strategy to address both animal and environmental reservoirs. This study, with its comprehensive collection of 141 *S*. Thompson from China, covering both clinical and non-human isolate sources over two decades, provides a foundation for in-depth exploration into the phylogeography, population genetics, and evolutionary dynamics of this serovar, particularly in isolates with the CIP^R^CTX^R^AZI^R^ phenotype.

The distinctive resistance profiles among various NTS serovars are mediated by either chromosomal mutations or horizontal gene transfer of various plasmids (58). Unlike other MDR serovars, such as Indiana/Kentucky, which harbor plasmid replicons like IncHI2/HI2A, IncX1, and IncI2 along with mutations in *gyrA* and *parC* (18, 59), the emergence of CIP^R^CTX^R^AZI^R^ phenotypes in *S*. Thompson is mainly due to a single IncC plasmid, which carries *qnrS1*, *qepA4*, *bla*_CMY-2_, and *mph*(A), mediating resistance to three front-line antimicrobial agents. This plasmid, previously identified in *E. coli* and *K. pneumoniae*, contained two MDR islands (ARI-A and ARI-B) and a main backbone typically housing the *bla*_CMY-2_ gene within the conjugative transfer region (60–62). In this study, IncC plasmids in CIP^R^CTX^R^AZI^R^ *S*. Thompson contains two large resistance regions, MDRR1 and MDRR2, the former of which is similar to the typically described ARI-B (61), alongside a *bla*_TEM-1B_ gene, and the latter of which carries *qnrS1*, *qepA4*, and *mph*(A) that has never been identified in other Enterobacterales. The integration of *qnrS1*, *qepA4*, and *mph*(A) into IncC plasmids has been previously observed separately through different arrangements of mobile genetic elements (63–65). However, the co-occurrence of these three genes in the same plasmid is rare. Therefore, the emergence of three ARGs, together with *bla*_CMY-2_ and other AGRs on a single IncC plasmid within *S*. Thompson, is of concern, as they mediate resistance to almost all antimicrobial agents for treating salmonellosis.

Plasmids often impose a fitness cost on their bacterial hosts (66). However, the IncC plasmid from CIP^R^CTX^R^AZI^R^ *S*. Thompson has demonstrated remarkable stability and specificity, exemplified by the clone transmission of IncC-carrying strains across various provinces in the south and east of China. Notably, although IncC plasmids have been detected in various *Salmonella* strains (67, 68), these IncC plasmids conferring CIP^R^CTX^R^AZI^R^ remain exclusive to this serovar (*S*. Thompson), further suggesting that the cross-regional spread of CIP^R^CTX^R^AZI^R^ *S*. Thompson is probably driven by the transmission of dominant adapted clones carrying the IncC plasmid. The emergence of lineage L6, harboring the IncC plasmids and conferring resistance to multiple antimicrobial agents, marks a significant evolutionary event, indicating a relatively recent and specific adaptation of clinically important plasmids within *S*. Thompson. Nonetheless, the key epidemiological drivers propelling the wide transmission of these clinically important clones remain elusive. The frequent identification of the virulence factor SspH1 within the L6 strain suggests its potential role in enhancing *Salmonella* infection, which may contribute to its increased clinical detection rates (69). However, further studies are needed to confirm whether this heightened presence is indeed due to the functional activities of SspH1. Furthermore, the predominance of clinically important clones in L6, primarily composed of isolates from both patients and aquatic products, hints at a connection with the trade in aquatic food products, which has been proven in previous research (57). In this study, the strains of L6 derived from aquatic products were mainly isolated from freshwater fish, and there is additional evidence that turtles are also reservoirs of *S*. Thompson (70–72). Notably, certain provinces in southern China have a dietary tradition of consuming soft-shelled turtles. Thus, the trade of aquatic products, particularly turtles, may play a role in the spread of this clinically important clone across various regions (73). Moreover, the presence of five clinical *S*. Thompson harboring the similarity IncC plasmid from USA and EU countries interspersed among Chinese isolates indicates the potential for interhost and international spreading.

The diversity in resistance phenotypes among *S*. Thompson may reflect host-specific factors and distinct antimicrobial selection pressures in various animal-derived food production settings (74). Over the past decade, intensive animal farming in China has witnessed a rapid increase, leading to the extensive use of antimicrobial compounds in livestock, poultry, and aquatic animal production (75, 76). Chinese aquaculture has utilized more than 20 different antimicrobials (77). Despite certain antimicrobial agents, such as chloramphenicol, ciprofloxacin, and erythromycin, being officially prohibited in aquaculture since 2002, their presence in high concentrations within aquaculture products indicates potential illicit application in this industry (77, 78). Notably, the majority of these antimicrobial compounds are not metabolized by aquatic organisms, rather, they enter the aquatic ecosystem as residues, intensifying the selective pressure on aquatic bacteria (79). For instance, aquatic environments can act as hotspots for transferable quinolone resistance, significantly exacerbating the spread of antibiotic resistance in aquatic ecosystems (80). Furthermore, ARGs and antimicrobial compounds from hospital wastewater, agricultural runoff, and urban sewage inevitably converge in these ecosystems, amplifying the challenge of AMR within the One Health framework (81, 82). These factors exert significant selective pressure on aquatic bacteria, doing some to facilitate the emerging and spreading of IncC-carrying CIP^R^CTX^R^AZI^R^ *S*. Thompson. The intricate web of antimicrobial usage and environmental contamination fuels the resistance crisis in terrestrial animal farming as well. For instance, the ARGs-carrying IncHI2/IncHI2A plasmid, identified in the chicken-derived *S*. Thompson in this study, is prevalent in multiple NTS (83, 84). Given the direct link between animal-derived foods and human health, the observed resistance patterns in *S*. Thompson pose a real threat by potentially enabling the direct transmission of these dominant clones from food supply to humans, thereby exacerbating the public health crisis related to AMR.

## Data Availability

The sequence data of all isolates have been submitted to NCBI under BioProject accession number PRJNA1204499.

## AFTER EPUB

*[This article was published on 12 February 2025 with errors in the presentation of figures in the PDF. The PDF was corrected in the current version, posted on 4 March 2025.]*

## SUPPLEMENTAL MATERIAL

Supplemental Figures msystems.01018-24-s0001.docx Fig. S1 to S5.

Supplemental Tables msystems.01018-24-s0002.xlsx Tables S1 to S7.

ASM does not own the copyrights to Supplemental Material that may be linked to, or accessed through, an article. The authors have granted ASM a non-exclusive, world-wide license to publish the Supplemental Material files. Please contact the corresponding author directly for reuse.
