# Supplementary material for: Tracing the evolution: the rise of Salmonella Thompson co-resistant to clinically important antibiotics in China, 1997–2020
Source: mSystems. 2025 Feb 12;10(3):e01018-24. doi: 10.1128/msystems.01018-24 (PMC11915813; doi:10.1128/msystems.01018-24)
Supplement: Supplemental Figures — Fig. S1 to S5. [file msystems.01018-24-s0001.docx]

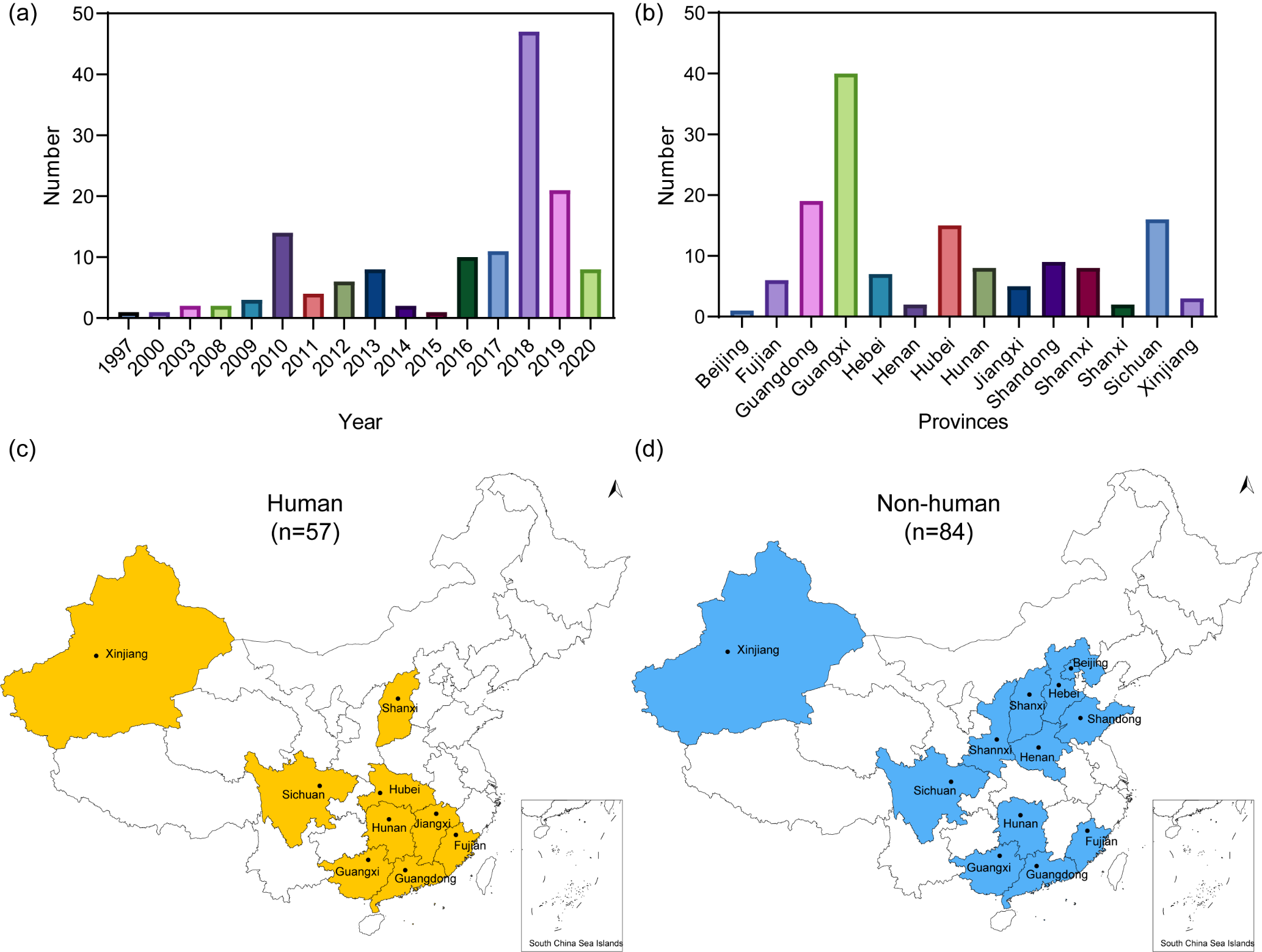


**Fig. S1** Metadata information describing all 141 *S*. Thompson. (a) Chronological distribution recording the isolation of the 141 *S*. Thompson; (b) Geographical origins of the 141 *S*. Thompson; (c) Geographical distribution of the 57 human-derived isolates; (d) Geographical distribution of the 84 non-human-derived isolates.

**
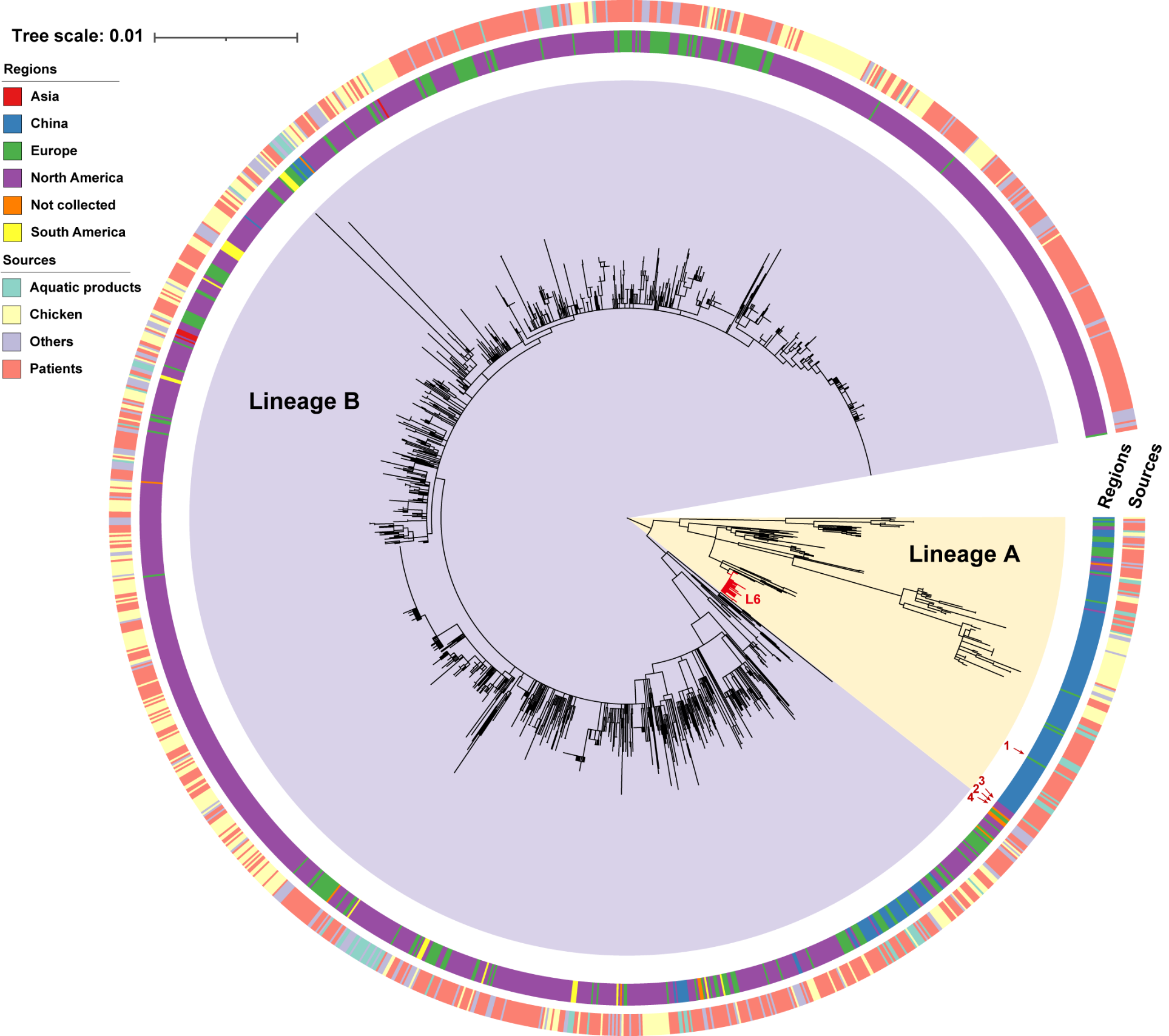
**

**Fig. S2** Comparative phylogenetic analysis of 141 local genomes with those of a further 1,484 online genomes international. The red branches represent the lineage 6 in this study, and the red arrows indicate the global strains that carry IncC: 1: United Kingdom: GCA 010794315.1, 2: USA: GCA 010585625.1, 3: USA: GCA 010494935.1, 4: USA: GCA 010727025.1.

**
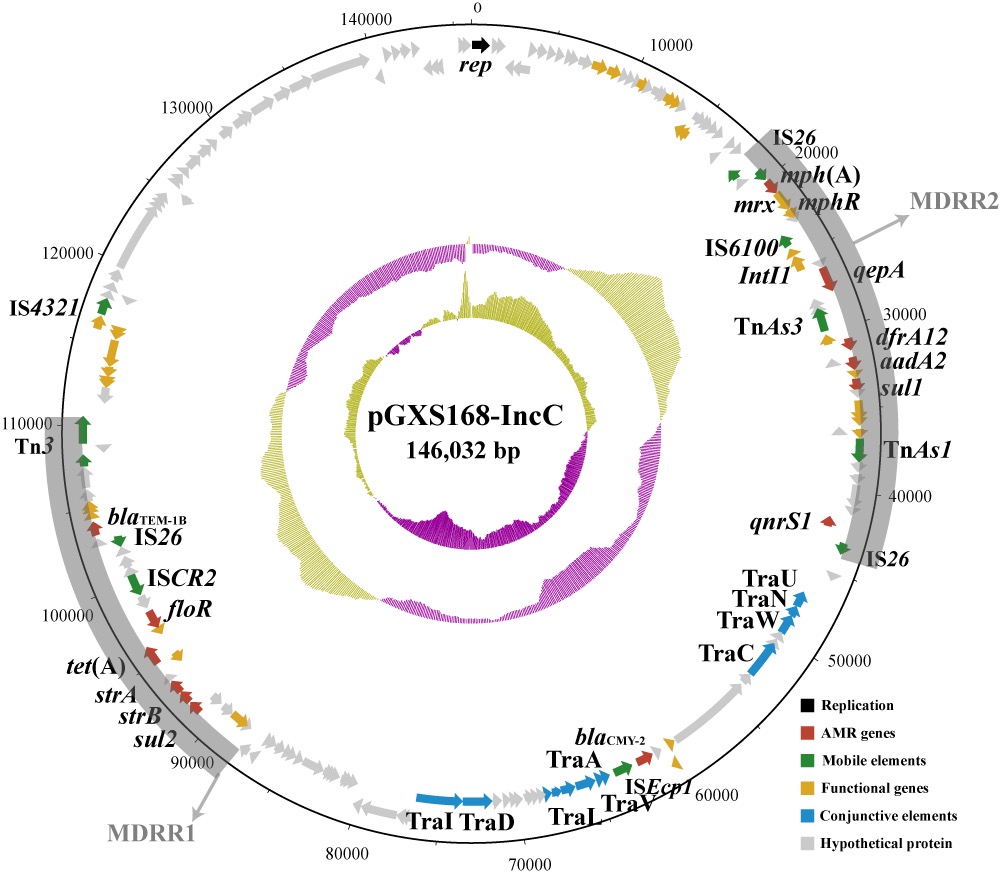
**

**Fig. S3** The detailed constructions of IncC-type plasmid pGXS168-IncC. The multidrug resistance regions (MDDRs) were highlighted in grey.


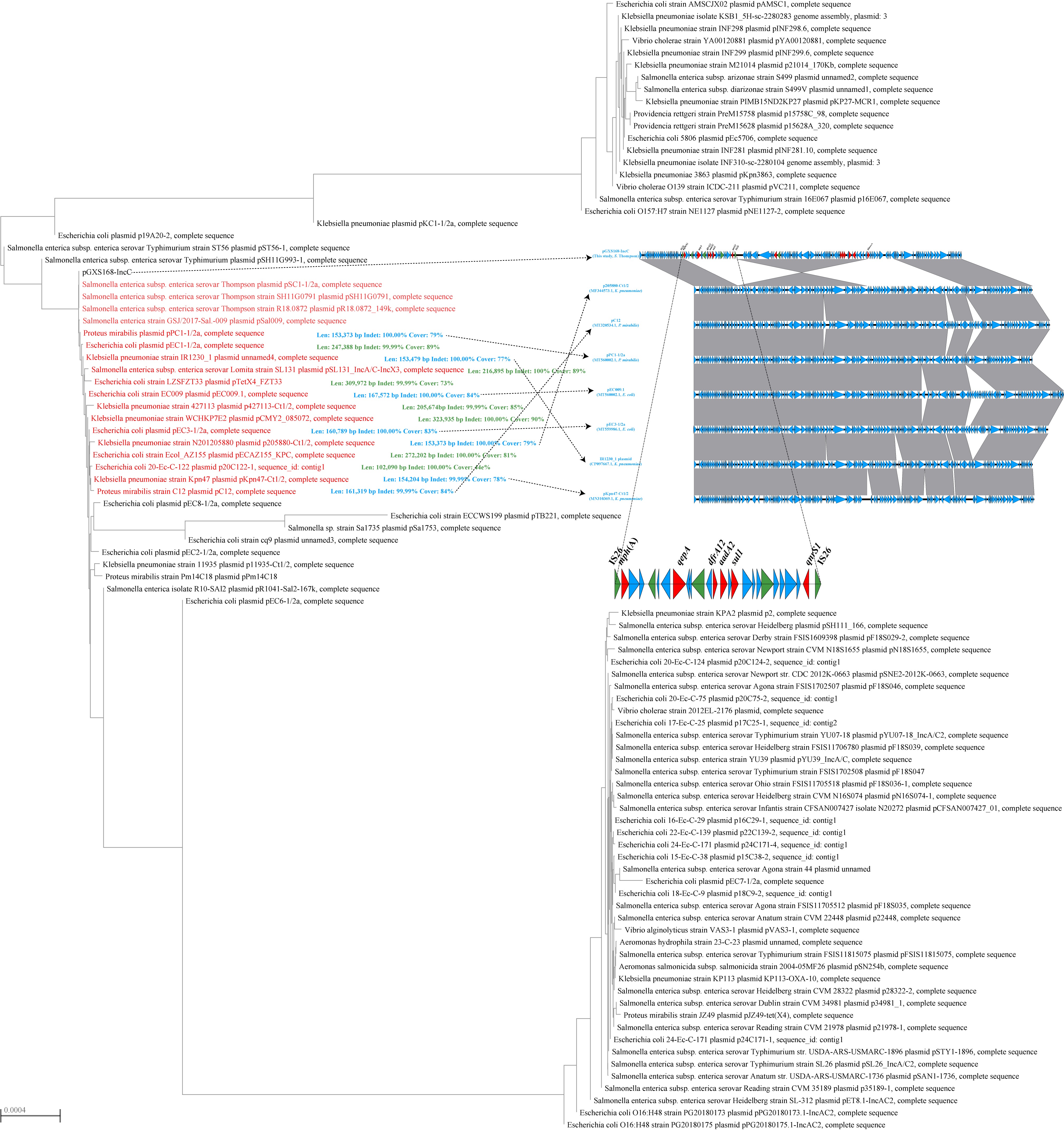


**Fig. S4** The phylogenetic analysis was performed using BLAST TREE (https://www.ncbi.nlm.nih.gov/blast/treeview). This tree was produced using BLAST pairwise alignments of pGXS168-IncC with nucleotide collection (nt) database (2023). Sequences displaying high sequence similarity (observed with 150-170 kbp of length and more than 75% coverage) was marked in blue. The MDRR2 was absent in these sequences with high similarities to pGXS168-IncC.


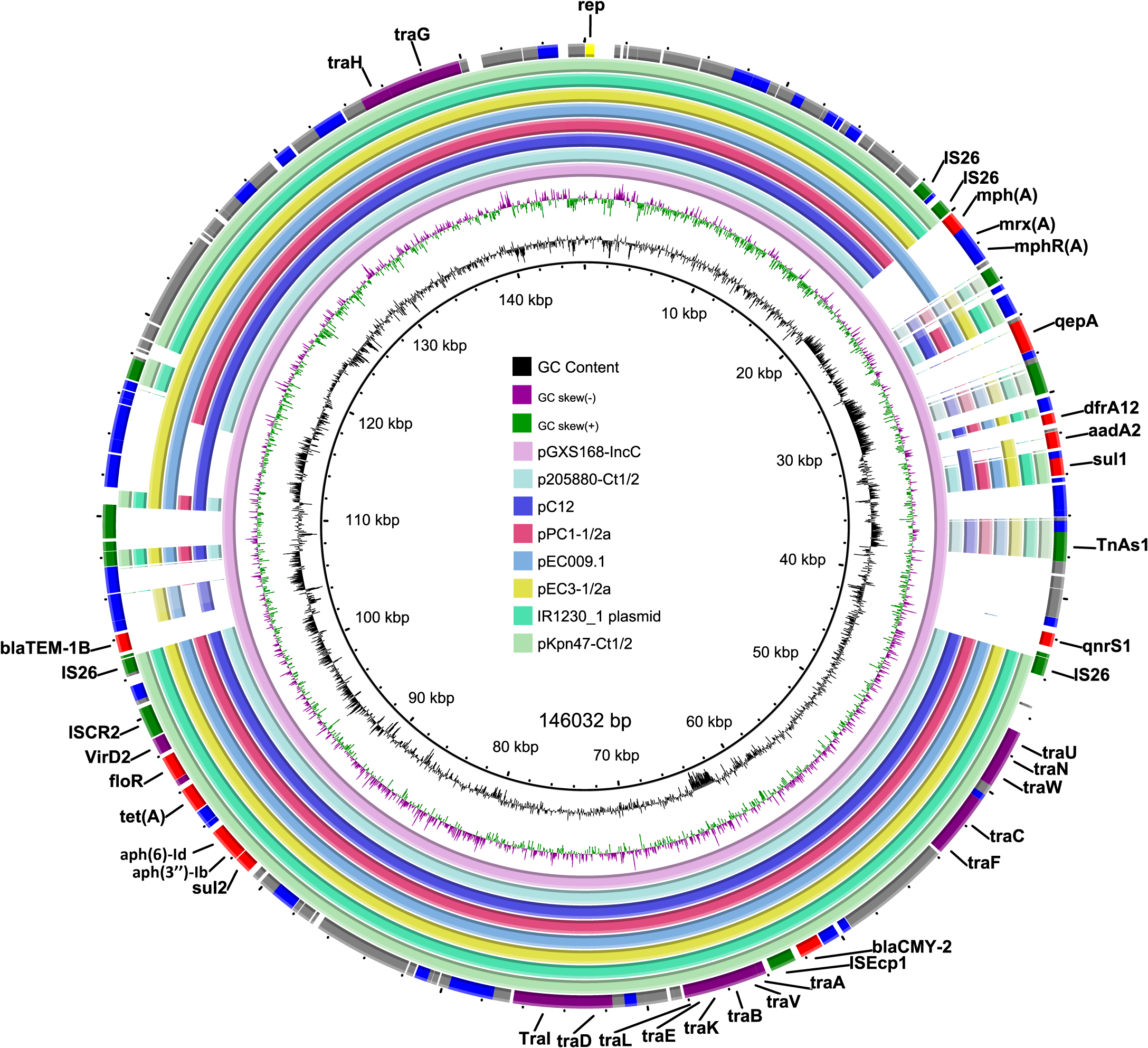


**Fig. S5 Multiple sequence alignment of pGXS168-IncC with seven publicly available plasmids.**
